# Oxycodone vs. tramadol in postoperative parent-controlled intravenous analgesia in children: a prospective, randomized, double-blinded, multiple-center clinical trial

**DOI:** 10.1186/s12871-023-02054-8

**Published:** 2023-05-03

**Authors:** Siyuan Li, Hongfei Xiong, Yingping Jia, Zhengchen Li, Yexi Chen, Liang Zhong, Feng Liu, Shuangquan Qu, Zhen Du, Yuxia Wang, Suxia Huang, Yonghui Zhao, Jing Liu, Lihua Jiang

**Affiliations:** 1 The Anesthesia & Comfort Health Center, Xi’an International Medical Center Hospital, Xi’an Shaanxxi, 710100 China; 2 Department of Anesthesiology, The Second Affiliated Hospital of Xian Jiaotong University, Xi’an, 710004 Shaanxi China; 3 Department of Anesthesiology, Children’s Hospital Affiliated of Zhengzhou University, Henan Children’s Hospital, Zhengzhou Children’s Hospital, Zhengzhou, 450007 China; 4 Department of Anesthesiology, Wuhan Children’s Hospital, Tongji Medical College, Huazhong University of Science & Technology, Wuhan, 430015 China; 5 Department of Anesthesiology, Hunan Children’s Hospital, Changsha, 410007 China; 6 Department of Anesthesiology, The Third Affiliated Hospital of Zhengzhou University, Zhengzhou, 450052 China; 7 Department of Anesthesiology, Zhumadian Central Hospital, Zhumadian, 463000 China; 8 The Second Affiliated Hospital of Xi’an Medical College, Xi’an, 710038 China

**Keywords:** Oxycodone, Tramadol, Parent-controlled intravenous analgesia, General anesthesia, Children

## Abstract

**Background:**

Management of acute postoperative pain is one of the major challenges in pediatric patients. Oral oxycodone has shown good pain relief in postoperative pain relief in children, but no studies have investigated intravenous oxycodone in this context.

**Objective:**

whether oxycodone PCIA can provide adequate and safe postoperative pain relief, in comparison to tramadol as reference opioid drug.

**Design:**

a randomized, double-blind, parallel, multi-center clinical trial.

**Setting:**

five university medical centers and three teaching hospitals in China.

**Participants:**

patients aged 3-month-old to 6-year-old undergoing elective surgery under general anesthesia.

**Intervention:**

patients were randomly allocated to either tramadol (n = 109) or oxycodone (n = 89) as main postoperative opioid analgesic. Tramadol or oxycodone were administered with a loading dose at the end of surgery (1 or 0.1 mg.kg^–1^, respectively), then with a parent-controlled intravenous device with fixed bolus doses only (0.5 or 0.05 mg.kg–1, respectively), and a 10-min lockout time.

**Outcomes:**

the primary outcome was adequate postoperative pain relief, defined as a face, legs, activity, cry, and consolability (FLACC) score < 4/10 in the post-anesthesia care unit (PACU), with no need for an alternative rescue analgesia. FLACC was measured 10 min after extubation then every 10 min until discharge from PACU. Analgesia was currently conducted with the boluses of either tramadol or oxycodone if FLACC was ≥ 3, up to three bolus doses, after what rescue alternative analgesia was administered.

**Results:**

tramadol and oxycodone provided a similar level of adequate postoperative pain relief in PACU and in the wards. No significant differences were either noted for the raw FLACC scores, the bolus dose demand in PACU, the time between the first bolus dose and discharge from PACU, analgesic drug consumption, bolus times required in the wards, function activity score, or the parents’ satisfaction. The main observed side effects in both groups were nausea and vomiting, with no difference between groups. However, patients in the oxycodone group showed less sedation levels and had a shorter stay in the PACU, compared with the tramadol group.

**Conclusions:**

an adequate postoperative analgesia can be achieved with intravenous oxycodone, this with less side effects than tramadol. It can therefore be a choice for postoperative pain relief in pediatric patients.

**Trial registration:**

The study was registered at www.chictr.org.cn (Registration number: ChiCTR1800016372; date of first registration: 28/05/2018; updated date:06/01/2023).

## Key points

Question: Can the intravenous administration of oxycodone provide adequate postoperative analgesia in children?

Findings: Oxycodone can provide well postoperative pain control in children, similar to tramadol.

Meanings: For the first time, we demonstrate that administration of oxycodone intravenously can provide well acute pain control in children after surgery, expanding our opioid drug choice for postoperative pain management in children patients.

## Background

Acute postoperative pain is one of the major challenges for the anesthesiologists [1]. A study in 2014 have shown that about 40% of American hospitalized children experienced moderate to severe pain, and 62-80% of children still experienced moderate or severe pain 48 h after neurosurgery when sufentanil or fentanyl was administrated for postoperative pain control [2], indicating insufficient postoperative analgesia for this group of children.

Compared to adults, children should receive analgesia through the most comfortable and least painful method available. The oral administration of analgesics is straightforward and has demonstrated good effectiveness in various settings. However, the oral route is often unsuitable for postoperative pain relief immediately after surgery due to the risk of nausea and vomiting or a slow return to normal gastric function [3]. As a result, patient-controlled analgesia (PCA) using continuous intravenous opioid infusions remains a highly effective method for managing acute postoperative pain in pediatric patients [4]. Recent evidence suggests that parent-controlled intravenous analgesia (PCIA) has some benefits over continuous opioid infusions [5, 6].

Morphine is the most widely utilized opioid in PCA for children in western countries and serves as the benchmark against which other analgesics are evaluated in clinical trials [4]. Other commonly used analgesics in PCA for children include sufentanil, fentanyl, and tramadol. Tramadol, a unique central-acting opioid, also possesses the ability to inhibit serotonin and noradrenaline receptors. Previous studies have shown that intravenous tramadol is equally effective as morphine in managing postoperative pain in children [7–10].

Oxycodone stands out among analgesics as a full agonist of both µ- and κ-opioid receptors [9, 10], offering superior pain relief for both somatic and visceral pain in adults [11, 12]. Its metabolism is more predictable and dosing is more manageable compared to morphine [13]. With a relatively long half-life of 5–6 h, oxycodone is suitable for PCA without the need for continuous infusion [13]. Despite its suitability as an analgesic for postoperative pain control in children, there is currently no research on the use of intravenous oxycodone for this purpose. However, oral administration of oxycodone has proven to be an effective means of managing acute pain in pediatric patients [14, 15].

In this study, we propose that oxycodone-based PCIA can provide postoperative pain relief in pediatric patients effectively and safely. We chose not to use morphine as a positive control due to its low acceptance rate of only 14.8% among pediatric patients in China, compared to higher rates for sufentanil (70.4%), fentanyl (66.7%), and tramadol (59.3%) [16]. Sufentanil, with its shorter duration of action, is not suitable for PCIA without continuous infusion. Fentanyl-based PCIA without continuous infusion, while reducing opioid consumption, may increase bolus times and complicate postoperative management for parents [17]. Tramadol, with its longer half-life and similar efficacy for postoperative pain relief compared to morphine [7–10], particularly in the post-anesthesia care unit (PACU) and 24 h post-operation [18], was chosen as the positive control drug in this study.

## Materials and methods

### Study design

This is a randomized, double-blind, parallel, multi-center clinical trial. This study was approved by the medical ethics committee, the second affiliated hospital of Xi’an Jiaotong University (2018024), and written informed consent was approved by each patient’s legal guardian. The study was registered at www.chictr.org.cn (Registration number: ChiCTR1800016372; Date of first registration: 28/05/2018). The registration information was updated on 06/Jan/2023 because of an English typing mistake, which caused a discrepancy in the Chinese version and the English in the inclusion criteria (e.g., ASA I-III in the Chinese version, and ASA I-II in the English version). The registration office has amended the English mistake after carefully checking all the original registration documents. We conducted a multi-center clinical trial in the Second Affiliated Hospital of Xi’an Jiaotong University (Xi’an, China), Henan Children’s Hospital & the Third Affiliated Hospital of Zhengzhou University (Zhengzhou, China), Wuhan Children’s Hospital, Tongji Medical College, Huazhong University of Science & Technology (Wuhan, China), Hunan Children’s Hospital (Changsha, China), Zhumadian Central Hospital (Zhumadian, China). The Good Clinical Practice guidelines and the guidelines of the Helsinki Declaration were followed in the study.

### Patients

Patients [3-month-old to 6-year-old, Body Mass Index (BMI) 18-29.5 kg/m^2^ and American Society of Anesthesiologists (ASA) I-III] undergoing major surgery including general, urinary, and orthopedic surgery under general anesthesia were recruited. Patients were excluded if they met any following criteria: schizophrenia, epilepsy, infantile autism, attention deficit hyperactivity disorder, myasthenia gravis, cerebral palsy, Down’s syndrome, coma, and any other brain injury or neurosurgery; taking monoamine oxidase inhibitors within 2 weeks before the surgery; children may ingested alcohol or narcotics for various reasons, including medication and family influence; diabetes; severe hepatic, renal, pulmonary dysfunction; congenital heart disease or other congenital malformation; prematurity; any other diseases potentially interfering study results; surgery shorter than one hour; incomplete case report form (CRF).

### Randomization and blinding

A biostatistician who is unaware of treatments and patient follow-up, generates the random numbers using the SAS software (SAS Institute, USA) in a ratio of 1:1, named group A and group B. The randomization sequence is stored online until the end of the study (https://pan.baidu.com). As new participants consented to join the study, the responsible anesthesiologist would contact the biostatistician, who would assign the participant to either group A or B based on the next unused random number from the online sequence. This allocation was marked as used in red.

Only the biostatistician was aware of the randomization sequence and did not participate in the conduct of the study or patient follow-up. The responsible anesthesiologist was aware of the drugs being used, oxycodone or tramadol, but was not involved in patient assignment, follow-up, or data analysis. The responsible anesthesiologist is aware of whether oxycodone or tramadol is used for the patients, however, he or she is not involved in the patient assignment, follow-up, and data analysis. The study drugs are prepared by the responsible anesthesiologist. The study drugs were prepared by the responsible anesthesiologist, diluted to the same volume with normal saline, and placed in similar PCIA devices without labeling. Throughout the study, all study personnel, patients, parents, and everyone except the responsible anesthesiologist, remained blind to the patients’ allocations.

In an emergency, each center’s principal investigator can request the treatment allocation unmasking. The case should be documented and analyzed to evaluate any association with the treatment.

### Procedures

Patients were fasting from clear liquids for 4 h and solid food for 6 h prior to undergoing anesthesia. No analgesic or antiemetic drugs were administrated before the procedure. Thirty minutes before the procedure, patients received an intramuscular administration of 0.01 mg.kg^–1^ atropine. Routine monitoring, including electrocardiogram (ECG), pulse oximetry (SpO_2_), heart rate (HR), noninvasive blood pressure (NBP), and bispectral index (BIS), was established before induction of general anesthesia, which was achieved through administration of 3 mg.kg^–1^ propofol, 0.2 mg.kg^–1^ cisatracurium, and 0.4 µg.kg^–1^ sufentanil. During the surgery, propofol (6-10 mg.kg^–1^.h^–1^) and remifentanil (0.1–0.3 µg.kg^–1^.min^–1^) were administered through continuous infusion, with adjustments maintaining BIS between 40 and 60 and blood pressure and/or heart rate alteration within 20% of baseline. Cisatracurium was maintained at a rate of 2 µg.kg^–1^.min^− 1^.

Cisatracurium infusion was discontinued 30 min prior to the end of surgery, and 0.5 mg.kg^–1^ ketorolac tromethamine, 0.1 mg.kg^–1^ dexamethasone, and 0.1 mg.kg^–1^ tropisetron were administrated intravenously. Upon completion of surgery, patients received intravenous administration of oxycodone (0.1 mg.kg^–1^) or tramadol (1.0 mg.kg^–1^) ^18^, followed by the administration of the PCIA without background infusion. The bolus dose was 0.05 mg.kg^–1^ for the oxycodone group and 0.5 mg.kg^–1^ for the tramadol group each time, with a lockout time of 10 min for all patients. Surgical time, consumption of sufentanil and remifentanil, incision length, and hemorrhage volume during surgery were recorded.

After surgery, all patients were transferred to the post-anesthesia care unit (PACU). Extubation was performed after full recovery of spontaneous ventilation and consciousness. Ten min after extubation, pain intensity was firstly evaluated using the face, legs, activity, cry, consolability (FLACC) scale [19], followed by repeated evaluation every 10 min until discharge from PACU. A bolus dose of oxycodone or tramadol was given to the patient immediately if the score was ≥ 4 according patient’s allocation. The procedures could be repeated after 10 min until the score < 4. If postoperative pain relief were not satisfactory after three continues bolus doses, an alternative rescue analgesia is administrated to the patients.

Postoperative follow-up inwards was performed by research personnel or a healthcare member who was blinded to the allocation of the patient. Postoperative intensity at rest was evaluated using the FLACC scale by the parents. A bolus dose was given to the patient by parents immediately if the FLACC score was above 3. All parents were instructed on how to use the FLACC scale to evaluate postoperative pain intensity by research personnel face to face until they completely understood the method. Pain intensity in response to turning around in bed was evaluated using the functional activity score (FAS) [20]. Briefly, 1point: no limitation means the patient’s activity is unrestricted by pain; 2 points: mild limitation means the patient’s activity is mild to moderately restricted by pain; 3 points: severe limitation means the patient’s ability to perform the activity is severely limited by pain. Bolus times, consumption of analgesic doses, and incidence of adverse events were also recorded. If postoperative pain relief were not satisfied after three continuous bolus doses, alternative rescue analgesia was administrated to the patients. The drug and dose used were decided by the responsible anesthesiologist. Parents’ s satisfaction scores for the pain relief were graded by the blinded parents according to the 5-point scale: very dissatisfied (1 point), dissatisfied (2 points), neither satisfied nor dissatisfied (3 points), satisfied (4 points), and very satisfied (5 points) [11, 21, 22]. The definition of hypoxemia was SpO_2_ < 90% (breathing room air) or < 92% (breathing supplemental O_2_).

### Outcomes

Outcome assessment was performed by researchers who were kept blind to the patient’s group assignments. The primary outcome was whether oxycodone could provide adequate postoperative pain relief after surgery in pediatric patients. The definition of adequate postoperative pain relief includes (1) the FLACC score< 4 in the PACU; (2) patients do not need alternatively rescue analgesia. Ten minutes after the extubation in the PACU, postoperative pain in PACU was firstly evaluated by research personnel unaware of the allocation of the patient according to the FLACC scale; the FLACC evaluation was repeated every 10 min until discharge from PACU. A bolus dose of oxycodone or tramadol is given to the patient immediately if the score ≥ 4 according to patient’s allocation. The procedures could be repeated after 10 min until the score ≤ 3. If postoperative pain relief is not satisfied after three continuous bolus doses, alternative rescue analgesia is administrated to the patient.

The secondary outcomes included No. of boluses administered and bolus-administered amount of opioid in PACU (equal to morphine) in PACU and in the wards, time spent from PACU discharge to the first bolus dose, bolus analgesia rate in the wards, FAS scores in the wards, parents’ satisfaction with the PCIA. We also recorded and analyzed the consciousness recovery time, extubation time, the length of staying PACU, Ramsay scores, and adverse events, especially hypoxemia.

### Statistical analysis

A pilot observational study in our center showed that the FLACC scores between the oxycodone and tramadol groups in PACU were 3.71 ± 1.71 and 4.49 ± 1.74, respectively. The sample size required was 166 patients (83 patients in each group) after calculation with two-tailed α = 0.05 and β = 0.2 using the SAS software (SAS Institute Inc., USA). Considering the possible dropout cases, we increased the sample size to 240 for initial enrollment.

GraphPad Prism 6.0 software (GraphPad Software Inc., USA) was used to perform the statistical analysis. The per-protocol analysis set consists of subjects who were randomly assigned to treatment, and have no major protocol violations such as violations of entry criteria, errors in treatment assignment and use of excluded medications. Gaussian distribution was tested using D’Agostino & Pearson analysis. Data was displayed as the median value (1st -3rd quartiles). An unpaired t test (if the data was normally distributed) or Mann-Whitney u test (if the data was not normally distributed) was used to analyze the numeric variables. FLACC scores, Ramsay sedation scores, and parent satisfaction scores were analyzed using a linear mixed-model to analyze the interaction and main effects of time and group. χ2 test or Fisher exact probability method (if the theoretical frequency was less than 5) was used to analyze the categorical variables were analyzed. A significant difference was defined as a value of P < 0.05.

## Results

**Fig. 1:**
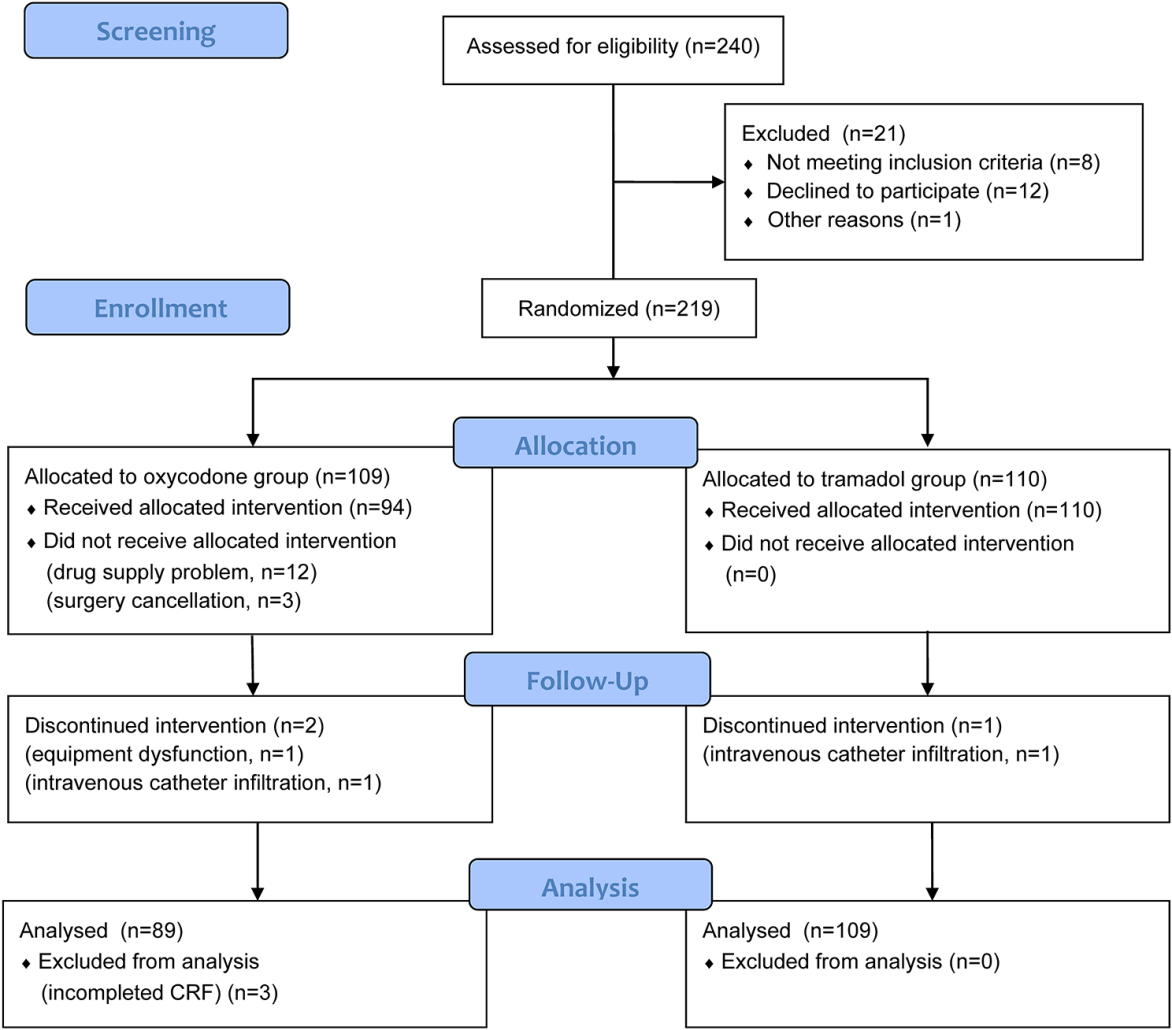
Trial profile. Data analysis included all patients in the groups to which they were randomly assigned. CRF: case report form

From October 2018 to September 2019, 240 patients were screened and 21 of them were excluded (Fig. 1). The remaining 219 patients were randomly assigned to either the oxycodone group (n = 109) or the tramadol group (n = 110). During the study period, a total of twenty patients dropped out from the oxycodone group and one from the tramadol group due to various reasons. Finally, data analysis was conducted on a total of 198 patients: 89 in the oxycodone group and 109 in the tramadol group.

We did not find any significant difference in the demographic data between oxycodone and tramadol groups (P > 0.05, Table 1).

**Table 1:** Comparison of demographic data between oxycodone & tramadol groups

Demographic data	Oxycodone (N = 89)	Tramadol (N = 109)	P
Female (%)	35 (39.3)	32 (29.4)	0.140
Age (year)	2.50 (1.15, 4.00)	2.40 (1.03, 4.00)	0.794
BMI (kg/m^2^)	16.5 (14.7, 19.1)	16.3 (15.2, 17.8)	0.564
ASA (I/II/III)	30/57/2	35/68/6	0.579
Surgery types (orthopedic/urinary/general/neuro/vascular)	16/33/35/3/2	24/32/48/2/3	0.571
Incision length (cm)	5.00 (3.00, 6.00)	4.00 (3.00, 6.00)	0.152
Duration of surgery (min)	110 (82.5, 200)	105 (75.0, 158)	0.215
Intraoperative remifentanil consumption (µg.kg^− 1^)	39.1 (25.3, 66.7)	35.7 (25.2, 46.4)	1.000
Intraoperative blood loss (ml)	12.0 (5.00, 30.0)	10.0 (5.00, 30.0)	0.613

Neither oxycodone nor tramadol group needed alternative rescue analgesia in PACU. FLACC scores at different time points in PACU were analyzed using a linear mixed-model. Most of the patients from the oxycodone and tramadol groups had well immediate postoperative pain relief (FLACC scores < 4) after extubation in PACU, and there was no significant difference in FLACC scores at 10 min, 20 or 30 min after extubation (P > 0.05, Table 2).No statistically difference was observed in the No. of boluses administered or bolus-administered amount of opioid (equal to morphine) in PACU between the oxycodone and tramadol groups (P > 0.05, Table 2).

After returning to the wards, all patients had adequate postoperative analgesia in the wards (FLACC scores < 4) after the bolus dose administration, and no alternative rescue analgesia was thereby recorded. There was no significant difference in the FAS scores (in response to turning around in bed), incidence rate of bolus analgesia, time spent from PACU discharge to the first bolus dose, No. of boluses administered, bolus-administered amount of opioid (equal to morphine) in the wards between the oxycodone and tramadol groups (P > 0.05, Table 2).

**Table 2:** Comparison the effect of postoperative pain relief between oxycodone & tramadol-PCIA

	Oxycodone (N = 89)	Tramadol (N = 109)	P
Pain scores in PACU (FLACC scale, 10min since extubation)	0 (0, 2.00)	0 (0, 2.00)	0.515
Pain scores in PACU (FLACC scale, 20min since extubation)	0 (0, 3.00)	0 (0, 2.25)	0.509
Pain scores in PACU (FLACC scale, 30min since extubation)	0 (0, 3.00)	0 (0, 2.50)	0.164
Functional activity scores in the wards (24h)	1.00 (1.00, 2.00)	1.00 (1.00, 2.00)	0.101
Functional activity scores in the wards (48h)	1.00 (1.00, 1.00)	1.00 (1.00, 1.00)	0.149
No. of boluses administered in PACU [n (%)]	22 (24.7)	25 (22.9)	0.769
Bolus-administered amount of opioid in PACU (equal to morphine, mg.kg^–1^)	0 (0, 0.050)	0 (0, 0.050)	0.640
Bolus analgesia in the wards [n (%)]	73 (82.0)	85 (78.0)	0.481
Time spent from PACU discharge to first bolus dose (min)	90.0 (33.0, 240)	137 (47.0, 315)	0.173
No. of boluses administered in the wards	5.00 (2.00, 10.0)	6.00 (2.50, 9.00)	0.775
Bolus-administered amount of opioid in the wards (equal to morphine, mg.kg^–1^)	0.370 (0.200, 0.630)	0.350 (0.210, 0.600)	0.259

PACU: postoperative anesthesia care unit; PCIA: parent control intravenous analgesia

**Fig. 2:**
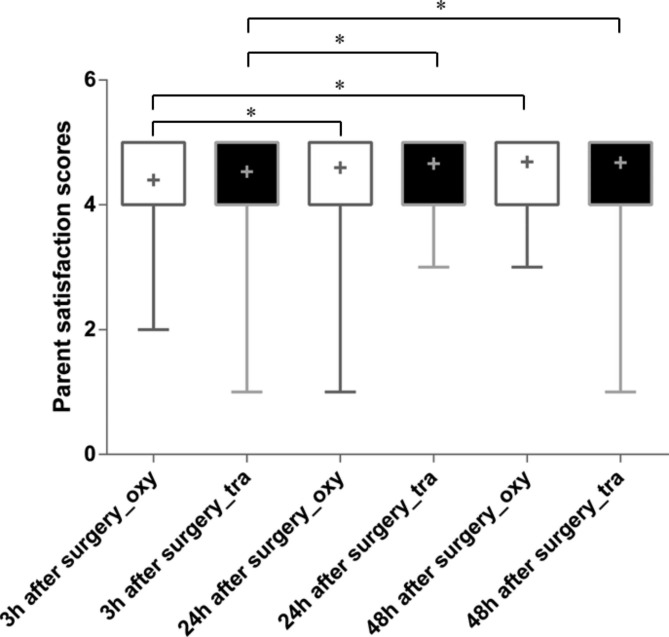
Comparison of parent satisfaction scores between oxycodone and tramadol group. * P < 0.05. +: mean; oxy: oxycodone group; tra: tramadol group

Parent satisfaction scores were analyzed using a linear mixed-model. There was no significant diffidence in parent satisfaction scores between the oxycodone and tramadol group at either 3 h (P = 0.553), 24 h (P = 0.553) or 48 h (P = 0.834) after surgery (Fig. 2). Compared with 3 h after surgery, higher parent satisfaction scores were observed at 24 h (P = 0.020) and 48 h after surgery (P < 0.001) in oxycodone group (Fig. 2). Similarly, higher parent satisfaction scores were observed at 24 h (P = 0.002) and 48 h after surgery (P = 0.002) in oxycodone group (Fig. 2). However, we did not observed difference in parent satisfaction scores between 24 and 48 h after surgery in either oxycodone (P = 0.064) nor tramadol group (P = 0.895).

During the anesthesia recovery period, there was no significant difference in the consciousness recovery time, extubation time, incidence of nausea & vomiting or pruritus between the oxycodone and tramadol groups (P > 0.05, Table 3). One patient in the tramadol group in PACU was observed hypoxemia with SpO_2_ recovering to the normal level after improving the patient’s body position, and naloxone was not needed. Patients in the tramadol group showed longer stays in the PACU than patients from the oxycodone group (Table 3).

**Table 3:** Comparison of recovery and adverse events between oxycodone and tramadol in PACU

Outcomes	Oxycodone (N = 89)	Tramadol (N = 109)	P
Consciousness recovery time (min)	18.0 (13.5, 37.5)	25.0 (15.0, 45.0)	0.078
Extubation time (min)	15.0 (12.0, 37.0)	20.0 (11.5, 39.0)	0.708
Nausea [n (%)]	1 (1.1)	2 (1.8)	1.000
Vomiting [n(%)]	0	0	--
Pruritus [n(%)]	2 (2.2)	1 (0.9)	0.589
hypoxemia [n(%)]	0 (0)	1 (0.9)	1.000
Length of stay in PACU (min)	28.0 (11.0, 40.0)	35.0 (15.0, 51.0)	0.008

PACU: postoperative anesthesia care unit

PCIA: parent control intravenous analgesia

**Fig. 3:**
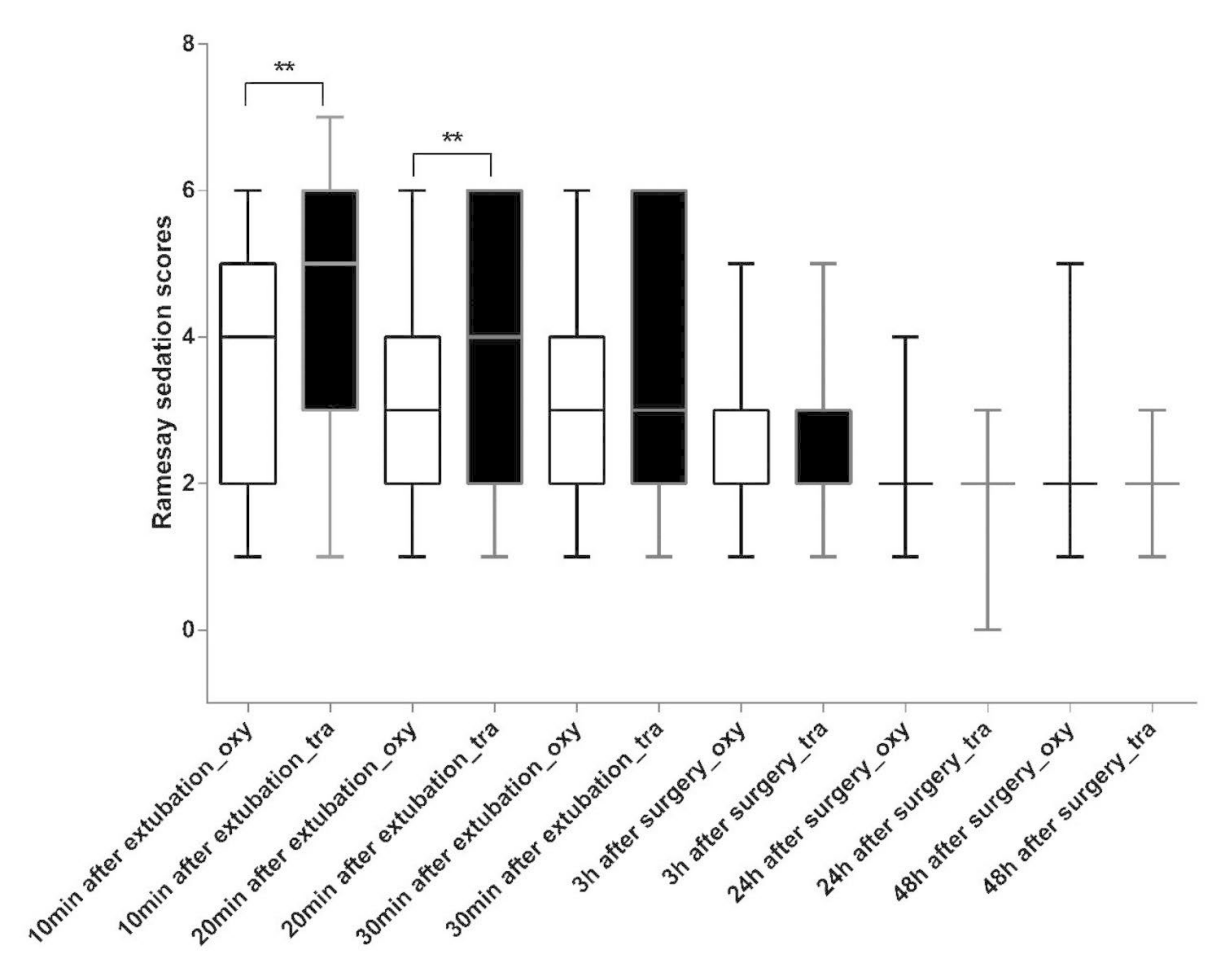
Comparison of Ramsay sedation scores between oxycodone and tramadol group. Patient′s sedation level in the PACU (10, 20 and 30 min after extubation) and in the wards (3 h, 24 and 48 h after surgery) was assessed by Ramsay sedation scores. ** P ≤ 0.01. oxy: oxycodone group; tra: tramadol group

Ramsay sedation scores were analyzed using a linear mixed-model. Patients from both oxycodone and tramadol groups showed a time-dependent reduction in Ramsay sedation scores in PACU (P < 0.05). Interestingly, patients from the oxycodone group showed significantly lower Ramsay sedation scores relative to the tramadol group 10 and 20 min after extubation in PACU (Fig. 3).

We further compared the other PCIA-related adverse events in the wards. As shown in Table 4, postoperative nausea, vomiting and pruritus were observed both in the oxycodone and tramadol groups, but we did not found significant difference in their incidence rate between these two groups (P > 0.05). No hypoxemia or over-sedation in patients from these two groups was observed in the ward.

**Table 4:** Comparison of adverse events between oxycodone and tramadol-PCIA in wards

Side effects	Time points	Oxycodone (N = 89)	Tramadol (N = 109)	P
nausea [n (%)]	3 h after surgery	5 (5.6)	3 (2.8)	0.471
	24 h after surgery	5 (5.6)	7 (6.4)	0.814
	48 h after surgery	5 (5.6)	8 (7.3)	0.627
vomiting [n (%)]	3 h after surgery	3 (3.4)	2 (1.8)	0.659
	24 h after surgery	3 (3.4)	7 (6.4)	0.330
	48 h after surgery	2 (2.2)	6 (5.5)	0.299
Pruritus [n (%)]	3 h after surgery	1 (1.1)	1 (0.9)	1.000
	24 h after surgery	2 (2.2)	1 (0.9)	0.589
	48 h after surgery	1 (1.1)	0	0.449
over sedation (Ramsay ≥ 5)	3 h after surgery	0	0	--
	24 h after surgery	0	0	--
	48 h after surgery	0	0	--
hypoxemia	3 h after surgery	0	0	--
	24 h after surgery	0	0	--
	48 h after surgery	0	0	--

## Discussion

There are several reasons for the cautious use of opioids in pediatric postoperative pain management, one of which is the fear of potential severe adverse effects due to children’s young age and small size. Although oral oxycodone has been widely used for pain relief in children, there is no literature on the use of intravenous oxycodone for postoperative pain relief in pediatric. In this study, we investigated whether intravenous oxycodone can provide adequate and safe postoperative pain relief, in comparison to intravenous tramadol which has a well-established use in children [18].

Oxycodone elimination is slower and its pharmacokinetics are more variable in neonates, particularly preterm newborns, and thus a lower dose with a longer dosing interval is recommended in these patients [23]. Previous studies have shown that children older than 6 months show similar pharmacokinetics of intravenous oxycodone to adults [23–25]. In this study, the youngest child recruited in the study was 6 months old.

Our study showed that children patients administrated with an equal dose of oxycodone or tramadol did not show any difference in the FLACC scores after extubation, the requirement for rescues analgesia in PACU. These results suggest that oxycodone could provide adequate transition analgesia compared with the equal dose of tramadol immediately after surgery, in line with previous studies conducted in adults [26–28]. Our findings also indicated that there was no difference in the recovery time from general anesthesia and extubation, implying that oxycodone and an equal dose of tramadol may have similar recovery of consciousness and cognition in children. The lower Ramsay score in the oxycodone group in the PACU may suggest that oxycodone has a less potent sedative effect than tramadol. This may explain the incidence of hypoxemia in the tramadol group in PACU, caused by oversedation, which was quickly resolved once the patient awakened and naloxone was not required. The varying levels of sedation in the PACU and ward may be due to differences in the conversion of oxycodone and tramadol dosages [26].

After returning to the wards, we did not record any requirements for the alternative rescue analgesia in either oxycodone or tramadol groups, suggesting that both oxycodone and tramadol-PCIA provide adequate postoperative pain control in children patients. Additionally, there was no significant difference in the bolus dose administration, including bolus dose times, the anesthetics consumption equivalent with morphine, the length between the first bolus dose and the discharge from PACU, and FAS scores between the two groups, demonstrating that oxycodone and tramadol have similar efficacy in providing postoperative analgesia in pediatric patients. Differently with the period of staying in PACU, we did not observe any cases of oversedation or significant differences in Ramsay scores between the oxycodone and tramadol groups in the ward. Considering the much lower dose administrated during PCIA infusion in the wards compared with the loading dose at the end of surgery, this result further illustrated that the higher sedation effect of tramadol relative to oxycodone observed in PCAU was dose-associated.

Our results align with previous studies that show that nausea and vomiting (PONV) is the most frequently reported adverse effect in both the oxycodone and tramadol groups [18, 26, 29, 30]. While the incidence of PONV in children treated with tramadol has been reported to be as high as 10%, our study showed a much lower incidence of less than 5%, likely due to our routine use of dexamethasone and tropisetron [31]. There is very limited data on postoperative oxycodone PCIA-related PONV in children. In a study performed in adults, the incidence rate of nausea and vomiting in patients was reported up to 48.4% and 12.5%, respectively [32]; however, the incidence rate can be reduced to < 10% after being given dexamethasone and tropisetron [11]. These results together demonstrate that the incidence rate of PONV could be reduced to an acceptable level by combinational use of glucocorticoids and 5-HT3 agonists, which may be an important advantage in the tramadol/oxycodone-based management of postoperative pain.

This study has several limitations that need to be considered: firstly, we administered only a single dose of either oxycodone or tramadol. In another preliminary study we performed, 0.05, 0.1, and 0.15 mg.kg^–1^ oxycodone were administrated intravenously to children after major surgery respectively, and 0.15 mg.kg^–1^ oxycodone showed superior pain relief to 0.1 mg.kg^–1^ oxycodone. Therefore, it is important to conduct further research to determine the efficacy and adverse effects of different doses of oxycodone in children after surgery to meet the needs and expectations of patients and their parents. Additionally, the sample size in this study is small, making it challenging to accurately compare the incidence of adverse effects between oxycodone and tramadol. It is premature to conclude that oxycodone is better tolerated than tramadol at a similar level of analgesia, as more research with a larger sample size is needed to assess the efficacy/intolerance balance in children between these two drugs. Thirdly, the administration of either oxycodone or tramadol via PCIA may introduce potential bias due to parents’ training and understanding of postoperative pain management and evaluation systems such as NRS and Ramsay sedation scale. Finally, we did not include morphine as a control group in this study due to its low usage as a postoperative analgesia among children patients in China (14.8%), with sufentanil (70.4%), fentanyl (66.7%), and tramadol (59.3%) being the most commonly used opioids for PCIA [16]. However, as morphine is considered the “gold standard for opioids,“ it would be interesting to compare its efficacy and safety with intravenous oxycodone in future studies.

## Conclusion

In conclusion, our findings indicate that an adequate postoperative analgesia can be achieved with intravenous oxycodone, this with less side effects than tramadol. It can therefore be a choice for postoperative pain relief in pediatric patients.

## Data Availability

The datasets used and/or analysed during the current study are available from the **c**orresponding author on reasonable request.

## Abbreviations

PCIA: Parent-Controlled Intravenous Analgesia
BMI: Body Mass Index
ASA: American Society of Anesthesiologists
CRF: Case Report Form
ECG: electrocardiogram
SpO_2_: pulse oximetry
HR: Heart Rate
NBP: Noninvasive Blood Pressure
BIS: Bispectral Index
PACU: Post-Anesthesia Care Unit
FLACC: face, legs, activity, cry, consolability
FAS: functional activity score

## References

[1] Gan TJ, Habib AS, Miller TE (2014). Incidence, patient satisfaction, and perceptions of post-surgical pain: results from a US national survey[J]. Curr Med Res Opin.

[2] Walker SM (2015). Pain after surgery in children: clinical recommendations[J]. Curr Opin Anaesthesiol.

[3] Puntillo F, Giglio M, Varrassi G (2021). The Routes of Administration for Acute Postoperative Pain Medication[J]. Pain Ther.

[4] McDonald AJ, Cooper MG (2001). Patient-controlled analgesia: an appropriate method of pain control in children[J]. Paediatr Drugs.

[5] Czarnecki ML, Hainsworth K, Simpson PM (2020). A pilot randomized controlled trial of Outcomes Associated with parent-nurse controlled Analgesia vs. continuous opioid infusion in the neonatal intensive care Unit[J]. Pain Manag Nurs.

[6] Kanagasundaram SA, Cooper MG, Lane LJ (1997). Nurse-controlled analgesia using a patient-controlled analgesia device: an alternative strategy in the management of severe cancer pain in children[J]. J Paediatr Child Health.

[7] Engelhardt T, Steel E, Johnston G (2003). Tramadol for pain relief in children undergoing tonsillectomy: a comparison with morphine[J]. Paediatr Anaesth.

[8] Hullett BJ, Chambers NA, Pascoe EM (2006). Tramadol vs morphine during adenotonsillectomy for obstructive sleep apnea in children[J]. Paediatr Anaesth.

[9] Ozalevli M, Unlugenc H, Tuncer U (2005). Comparison of morphine and tramadol by patient-controlled analgesia for postoperative analgesia after tonsillectomy in children[J]. Paediatr Anaesth.

[10] Minkowitz H, Salazar H, Leiman D (2020). Intravenous tramadol is effective in the Management of Postoperative Pain following Abdominoplasty: A Three-Arm Randomized Placebo- and active-controlled Trial[J]. Drugs R D.

[11] Han L, Su Y, Xiong H (2018). Oxycodone versus sufentanil in adult patient-controlled intravenous analgesia after abdominal surgery: a prospective, randomized, double-blinded, multiple-center clinical trial[J]. Med (Baltim).

[12] Dang SJ, Li RL, Wang J (2020). Oxycodone vs Sufentanil in patient-controlled intravenous Analgesia after Gynecological Tumor Operation: a Randomized double-blind clinical Trial[J]. J Pain Res.

[13] Ordonez GA, Gonzalez BM, Espinosa AE (2007). Oxycodone: a pharmacological and clinical review[J]. Clin Transl Oncol.

[14] Yang YT, Chen B, Bennett CL. FDA Approval of Extended-Release Oxycodone for Children With Severe Pain[J].Pediatrics, 2016,137(5). 10.1542/peds.2016-0205 27244829

[15] Groenewald CB, Rabbitts JA, Gebert JT (2016). Trends in opioid prescriptions among children and adolescents in the United States: a nationally representative study from 1996 to 2012[J]. Pain.

[16] Tan L, Huang K, Zhao Y (2014). A survey of postoperative pain treatment for children in chinese tertiary general or children’s hospitals[J]. Int J Anesthesiology Resusc.

[17] Jung H, Lee KH, Jeong Y (2020). Effect of fentanyl-based intravenous patient-controlled analgesia with and without basal infusion on postoperative opioid consumption and opioid-related side Effects: a retrospective cohort Study[J]. J Pain Res.

[18] Schnabel A, Reichl SU, Meyer-Friessem C et al. Tramadol for postoperative pain treatment in children[J].Cochrane Database Syst Rev, 2015(3):D9574. 10.1002/14651858.CD009574.pub2 PMC6464560 25785365

[19] Crellin DJ, Harrison D, Santamaria N (2015). Systematic review of the Face, Legs, Activity, Cry and Consolability scale for assessing pain in infants and children: is it reliable, valid, and feasible for use?[J]. Pain.

[20] Levy N, Sturgess J, Mills P (2018). Pain as the fifth vital sign” and dependence on the “numerical pain scale” is being abandoned in the US: why?[J]. Br J Anaesth.

[21] Di Palo MT (1997). Rating satisfaction research: is it poor, fair, good, very good, or excellent?[J]. Arthritis Care Res.

[22] Farooq F, Khan R, Ahmed A (2016). Assessment of patient satisfaction with acute pain management service: monitoring quality of care in clinical setting[J]. Indian J Anaesth.

[23] Kokki M, Heikkinen M, Valitalo P (2017). Maturation of oxycodone pharmacokinetics in neonates and infants: Oxycodone and its metabolites in plasma and urine[J]. Br J Clin Pharmacol.

[24] Kokki H, Rasanen I, Reinikainen M (2004). Pharmacokinetics of oxycodone after intravenous, buccal, intramuscular and gastric administration in children[J]. Clin Pharmacokinet.

[25] El-Tahtawy A, Kokki H, Reidenberg BE (2006). Population pharmacokinetics of oxycodone in children 6 months to 7 years old[J]. J Clin Pharmacol.

[26] Silvasti M, Tarkkila P, Tuominen M (1999). Efficacy and side effects of tramadol versus oxycodone for patient-controlled analgesia after maxillofacial surgery[J]. Eur J Anaesthesiol.

[27] Kampe S, Wolter K, Warm M (2009). Clinical equivalence of controlled-release oxycodone 20 mg and controlled-release tramadol 200 mg after surgery for breast cancer[J]. Pharmacology.

[28] Van Brussel C, Delefortrie Q, Kerzmann B (2017). [Oxynorm(R) instant versus tradonal(R) odis as level 2 analgesic in an emergency service: a monocentric double blind randomized non-inferiority study][J]. Rev Med Liege.

[29] Xie K, Zhang W, Fang W (2017). The analgesic efficacy of oxycodone hydrochloride versus fentanyl during outpatient artificial abortion operation: a randomized trial[J]. Med (Baltim).

[30] Finkel JC, Rose JB, Schmitz ML (2002). An evaluation of the efficacy and tolerability of oral tramadol hydrochloride tablets for the treatment of postsurgical pain in children[J]. Anesth Analg.

[31] Holt R, Rask P, Coulthard KP (2000). Tropisetron plus dexamethasone is more effective than tropisetron alone for the prevention of postoperative nausea and vomiting in children undergoing tonsillectomy[J]. Paediatr Anaesth.

[32] Kim NS, Lee JS, Park SY (2017). Oxycodone versus fentanyl for intravenous patient-controlled analgesia after laparoscopic supracervical hysterectomy: a prospective, randomized, double-blind study[J]. Med (Baltim).

